# Three cases of imported eyeworm infection in dogs: a new threat for the United Kingdom

**DOI:** 10.1136/vr.104378

**Published:** 2017-09-28

**Authors:** John Graham-Brown, Paul Gilmore, Vito Colella, Lyndsay Moss, Chris Dixon, Martin Andrews, Peter Arbeid, Jackie Barber, Dorina Timofte, John McGarry, Domenico Otranto, Diana Williams

**Affiliations:** 1 Liverpool Veterinary Parasitology Diagnostics, University of Liverpool, Liverpool, UK; 2 Institute of Infection and Global Health, University of Liverpool, Liverpool, UK; 3 Faculty of Veterinary Medicine, University of Bari, Bari, Italy; 4 Veterinary Vision Ltd Ophthalmic Referrals, Penrith, UK; 5 Cedar Veterinary Group, Alton, UK; 6 Kynance Veterinary Clinic, London, UK; 7 Institute of Veterinary Science, University of Liverpool, Neston, UK; 8 Pathology and Paraclinical Department, Institute of Veterinary Science, University of Liverpool, Liverpool, UK

**Keywords:** parasitology, vector-borne diseases, zoonoses, pets, legislation

## Abstract

In July 2016 we described the first known case of canine ocular thelaziosis in the UK in a dog recently imported from Romania. Here we confirm our initial diagnosis using PCR followed by sequence analysis, and we report a further two clinical cases in dogs with recent history of travel to Italy and France. In view of the presence in the UK of the vector for *Thelazia callipaeda*, namely *Phortica* spp, we discuss the significance of these three cases in the context of the UK government’s pet travel scheme, disease control and both animal and public health in the UK.

## Introduction

*Thelazia callipaeda* (Spirurida, Thelaziidae) is a vectorborne, zoonotic nematode capable of infecting a range of mammalian host species including dogs, cats and human beings, as well as several sylvatic species.^1^ The risks of introducing such parasitic agents to the UK posed by importation and/or travel of dogs abroad have been raised and illustrated on multiple occasions,^2–4^ while widespread media reports of large-scale illegal importation of dogs to the UK are clearly also of concern.

Adult *T callipaeda* reside in the eyes and associated tissues, including the conjunctival fornices, nictitating membrane, sclera and cornea, and the lacrimal glands of the definitive host.^5^ Infected animals show a variety of clinical presentations, from subclinical carriage through to mild (eg, epiphora, conjunctivitis and chemosis) and severe pathology including corneal ulceration, which, if untreated, can lead to complications including secondary infections and blindness. *T callipaeda* is sensitive to both milbemycin and moxidectin, with commercially available oral and spot-on applications shown to be effective treatment options.^6–8^

*T callipaeda*, also referred to as ‘the oriental eye worm’, is increasingly common in Europe, with autochthonous transmission confirmed in Italy, France, Switzerland, Germany, Spain, Portugal, Bosnia and Herzegovina, Croatia, Romania, Bulgaria, Hungary and Greece,^9–18^ with further cases reported in Belgium and Serbia.^7 19^ In some locations, such as the Basilicata region of Italy, infection is hyperendemic, with the reported prevalence in dogs exceeding 40 per cent.^20^ Furthermore, cases of human ocular thelaziosis in Spain, Italy, France, Croatia and Serbia demonstrate the parasite’s zoonotic potential.^19 21–23^

The intermediate host of *T callipaeda* in Europe has been identified as the male drosophilid fruit fly *Phortica variegata.* 24 These flies are both anthropophilic and zoophilic, transmitting the infective larval stages while feeding on lacrimal secretions.^25^ Studies have shown these flies are generally located in areas of oak woodland, with peak activity at 20°C–25°C and 50–75 per cent relative humidity.^26^ Development within the intermediate host may, under optimal conditions, be as short as 14 days,^25^ which combined with a prepatent period of four to eight weeks in the definitive host means that the peak transmission of *T callipaeda* is typically late summer/early autumn in Mediterranean countries.^5 26^

The UK government’s pet travel scheme (PETS) facilitates the travel of dogs to and from countries in the EU without the need for quarantine. In its current form owners are required to fulfil a number of specific requirements before and during travel abroad in order to ensure the implementation and documentation of control measures designed to prevent the importation of non-endemic zoonotic pathogens, specifically rabies and *Echinococcus multilocularis* (https://www.gov.uk/take-pet-abroad). Given the relatively free and regular movement of dogs into and out of the UK from mainland Europe and importation from rescue charities under this scheme, other pathogens, including *T callipaeda*, pose a significant threat to the UK canine population. The following case reports highlight the clinical, epidemiological and public health aspects of this disease and the implications for UK animal and human populations.

## Case 1

A one-year-old male collie cross (17 kg) was admitted for neutering on March 24, 2016. While under general anaesthesia, nematodes (n=10) were noted on the conjunctiva of both eyes and were removed using atraumatic forceps. The worms were not associated with pathological changes and no other clinical abnormalities were detected. Before discharge, the dog was given a single treatment with imidacloprid and moxidectin (100 and 25 mg, respectively, Advocate Spot-On Solution for Medium Dogs; Bayer), which are known to be effective for ocular thelaziosis.^7^ Five nematodes were placed in a 10 per cent formalin solution and submitted to Liverpool Veterinary Parasitology Diagnostics (LVPD) for identification (see below). No further parasites or ocular disease were detected at the five-day postoperative follow-up appointment.

## Case 2

A 12-year-old female wire hair fox terrier (9.5 kg) presented to its local (UK-based) veterinary surgery on September 21, 2016 suffering from conjunctivitis of the left eye. The dog had been seen 20 days previously for this complaint by a veterinarian in Italy when a topical course of chloromycetin (unspecified) had been prescribed. On clinical examination worms were detected on the conjunctiva of the affected eye and the animal was admitted for further examination. Following sedation with medetomidine and butorphanol (0.24 mg, Domitor 1 mg/ml solution for injection, Vetoquinol UK; and 0.95 mg, Torbugesic 10 mg/ml solution for injection, Zoetis UK, respectively), approximately 10 nematodes were manually recovered from beneath the nictitating membrane and conjunctival sac, following which the sedation was reversed with atipamezole (125 µg/kg, Antisedan 5 mg/ml solution for injection; Zoetis UK). Based on the suspicion of ocular thelaziosis,^6^ an oral dose of milbemycin and praziquantel (5 and 50 mg, respectively, Milbemax tablets for small dogs and puppies; Novartis Animal Health UK) was administered, and topical fusidic acid (one drop twice daily, Isathal 10 mg/g eye drops; Dechra Veterinary Products) was prescribed with a seven-day course for the affected left eye. Two nematodes were placed in physiological saline and submitted to LVPD via Axiom Laboratories (Newton Abbot, Devon, UK) for identification (see below). No further worms were detected at a seven-day follow-up appointment. At this time the topical course of fusidic acid course was extended for a further seven days, and an additional treatment with imidacloprid and moxidectin (100 and 25 mg, respectively, Advocate Spot-On Solution for Medium Dogs; Bayer) was also administered. No further problems have been reported since.

## Case 3

An eight-year-old neutered female West Highland white terrier (8 kg) was presented on October 31, 2016 with superficial corneal ulceration of the right eye of a reported duration of one week. The dog was otherwise clinically healthy. A seven-day course of topical chloramphenicol 1.0 per cent w/w eye ointment (5 mm three times daily; Martindale Pharmaceuticals) and oral meloxicam (0.1 mg/kg once daily, Metacam 1.5 mg/ml oral suspension for dogs; Boehringer Ingelheim Vetmedica) was prescribed initially. At seven days corneal ulceration had failed to re-epithelialise and the decision was made to refer the case to a dedicated ophthalmology centre for further investigation (Veterinary Vision, Cumbria).

**FIG 1:**
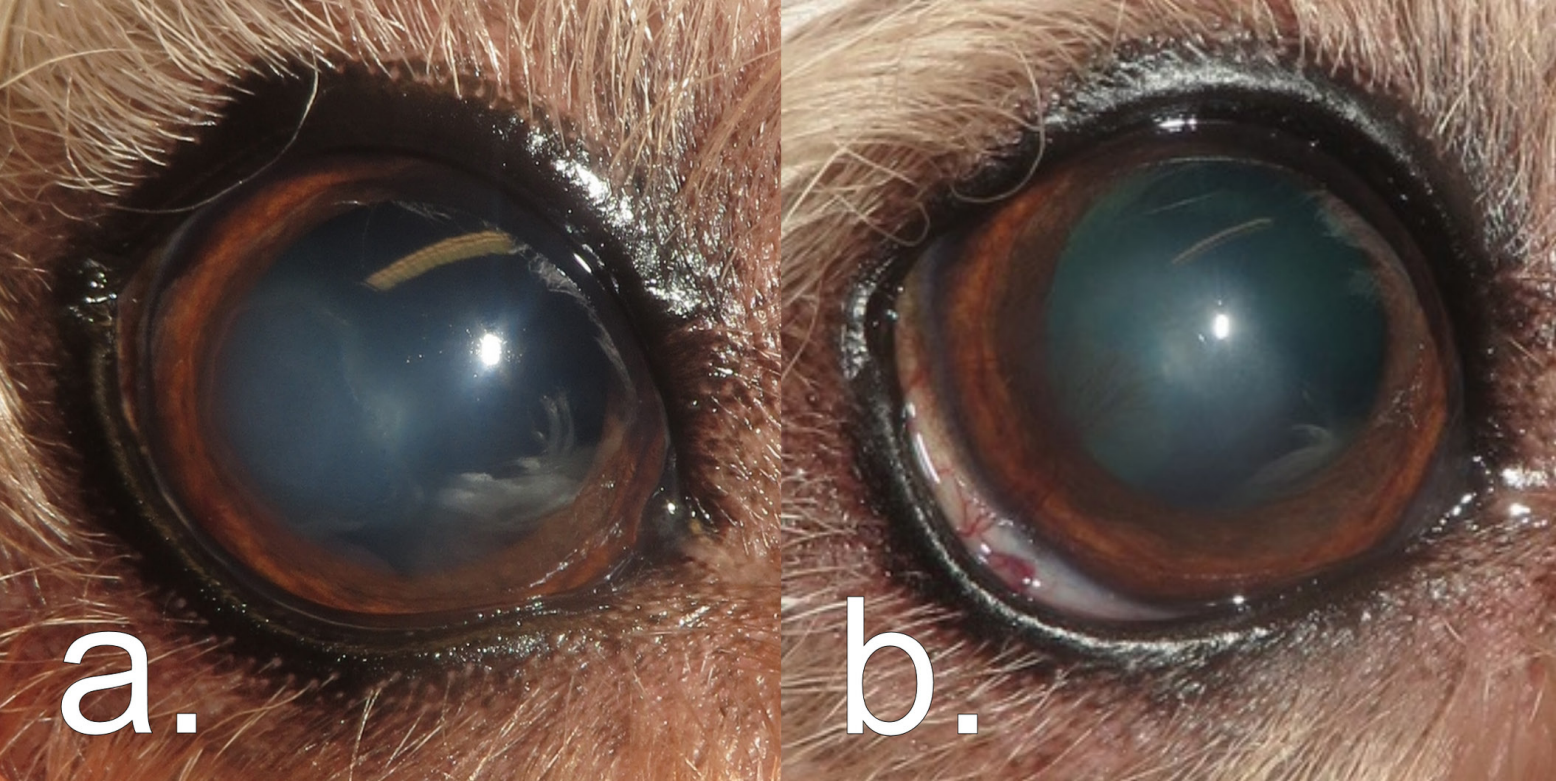
*Thelazia callipaeda*-associated pathology in the right eye of case 3 showing (a) superficial ventrolateral corneal ulceration at the initial point of referral, and (b) re-epithelialisation associated with ventral bulbar conjunctival hyperaemia, ventrolateral corneal oedema and ventrolateral superficial corneal vascularisation 21 days after flushing and removal of a single male *T callipaeda* specimen from the ventral conjunctival fornix.

Examination with Veterinary Vision on November 9, 2016 revealed right serous ocular discharge and increased blink rate indicating ocular discomfort. A Schirmer tear test (MSD Animal Health) confirmed increased lacrimation in the affected eye measuring 25 and 18 mm/minute on the right and left, respectively. Right conjunctival hyperaemia, ventrolateral corneal oedema and superficial corneal vascularisation were detected with slit lamp biomicroscopy (Keeler PSL Classic; Keeler), with a ventrolateral break in the corneal epithelium observed (Fig 1a). The presence of superficial corneal ulceration was confirmed by topical application of fluorescein stain (Fluorescein Sodium Ophthalmic Strips USP). No ectopic cilia, distichia or foreign bodies were visualised, which could have resulted in corneal abrasion. Following topical application of proxymetacaine hydrochloride (one drop, Minims Proxymetacaine Hydrochloride 0.5 per cent w/v Eye Drop Solution; Bausch and Lomb), intraocular pressure (measured with a Tono-Pen Avia Vet; Reichert Technologies) was recorded as within normal reference range bilaterally.^27^ No abnormalities of the eyelids, uveal tract, intraocular lens or fundus were identified. No abnormalities of the left eye were detected.

Irrigation of the conjunctival fornices with sterile water (Sterilised Water for Injections BP; Dechra Veterinary Products) yielded a single worm, flushed onto the corneal surface from the ventral fornix. The worm was removed with a sterile cotton bud, placed in 0.9 per cent sodium chloride (Aqupharm 1 Sodium Chloride 0.9 per cent w/v; Animalcare) and submitted to the University of Liverpool for identification. A course of topical ofloxacin (one drop four times daily, Exocin 0.3 per cent w/v eye drops 5 ml; Allergan) and hyaluronic acid (one drop four times daily, Remend Corneal Gel; Bayer) was prescribed for the affected right eye and oral meloxicam once daily continued for a further seven days.

Re-examination on November 30, 2016 (21 days post-treatment) showed a significant improvement in ocular comfort with a regular blink rate and lack of ocular discharge. Slit lamp biomicroscopy revealed right ventral bulbar conjunctival hyperaemia, ventrolateral corneal oedema and ventrolateral superficial corneal vascularisation (Fig 1b). Complete re-epithelialisation of the ulcer was confirmed using fluorescein stain. No further worms were identified by examination or flushing of the eyes or nasolacrimal ducts. A four-week course of topical dexamethasone (one drop twice daily, Maxitrol eye drop suspension 0.1 per cent; Alcon Pharmaceutical Products) was prescribed for the right eye, and treatment with imidacloprid and moxidectin (40 and 10 mg, respectively, Advocate Spot-On Solution for Small Dogs; Bayer) was advised for what was suspected to be a case of ocular thelaziosis.^8^ No further problems have been reported since.

## Travel history

All three cases had a history of recent time abroad in countries where *T callipaeda* is known to be endemic. The dog in case 1 had been imported to the UK from western Romania six weeks previously, having been initially found in Hateg, Hunedoara (45.61 N, 22.95 E). This location is geographically consistent with previous autochthonous *T callipaeda* case reports in both domestic dogs and sylvatic species.^28 29^

The dog in case 2 had recently returned from travel to northern Italy, having resided at a rural location close to Milan, Lombardia (45.48 N, 9.85 E). *T callipaeda* is well established across the north of Italy, with hyperendemicity in the adjoining region of Piedmont (North West), where prevalence in dogs has been previously reported at 23.1 per cent.^20 26^

The dog in case 3 had spent one month in France from August to September 2016, having never previously travelled outside the UK. This travel included time in Saint Avit Loisirs, Dordogne (44.95 N, 0.85 E). This location is less than 10 km from the site where autochthonous transmission of *T callipaeda* was first reported in France,^10^ with a more recent study identifying Dordogne as a focal point for *T callipaeda* in southern France.^30^

In all three cases, all requirements specified under PETS were met before and during travel, including microchipping, rabies vaccination and a tapeworm treatment administered by a veterinary surgeon 24–120 hours before (re-)entering the UK. In all cases tapeworm treatment consisted of a single dose of oral praziquantel as part of a combination dewormer also containing febantel and pyrantel (5, 15 and 14.4 mg/kg, respectively; Drontal Plus Tablets; Bayer). In case 3, the owners also administered their routine wormer (piperazine 937.5 mg and dichlorophen 1875 mg orally; Beaphar UK) and a generic shop-bought flea treatment (unspecified spot-on) following re-entry into the UK.

## Morphological identification

Specimens received by LVPD were either mounted in lactophenol, methylene blue clearing solution for morphological identification or preserved in 70 per cent ethanol for molecular analysis. Specimens from all three cases were examined by light microscopy (20–40× magnification) and identified as *T callipaeda* based on the key diagnostic morphological features described by Otranto *et al*. (2003).^31^

**FIG 2:**
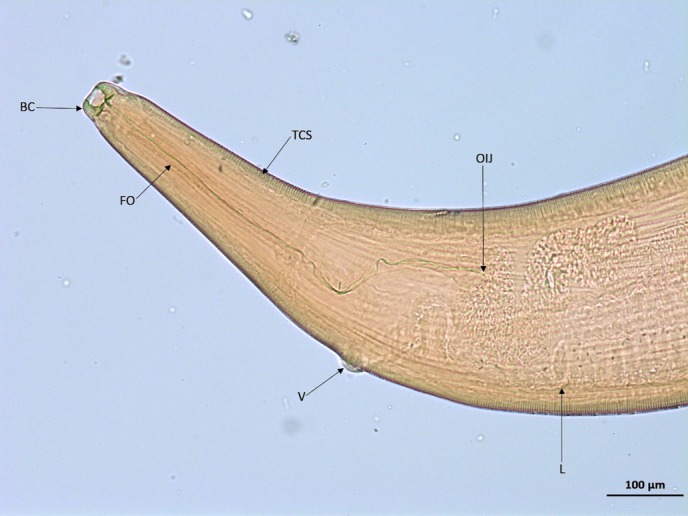
Light micrograph of female *Thelazia callipaeda*, anterior. BC, buccal capsule; FO, filariform oesophagus; L, L1 larvae; OIJ, oesophagus–intestinal junction; TCS, transverse cuticular striations; V, vulva (×10).

**FIG 3:**
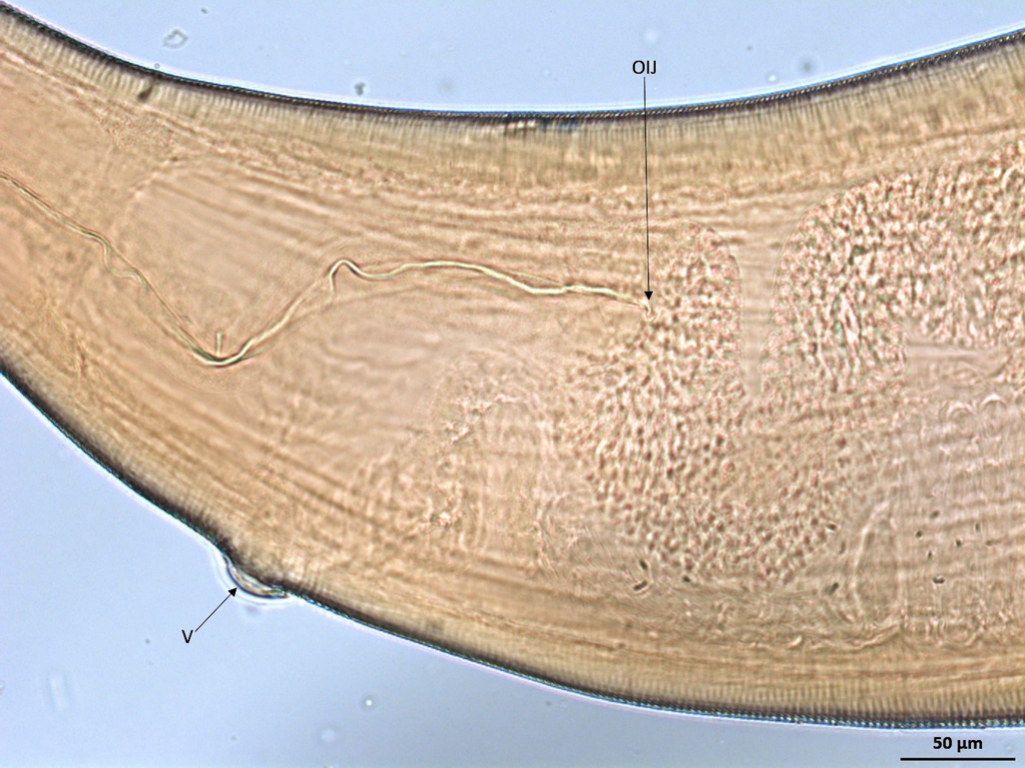
Light micrograph of female *Thelazia callipaeda*, anterior. Vulva (V) positioned anterior to the oesophagus–intestinal junction (OIJ) (×20).

**FIG 4:**
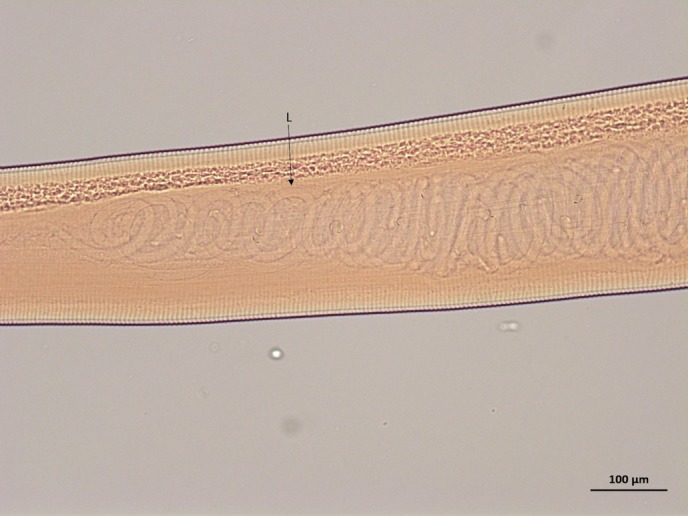
Light micrograph of female *Thelazia callipaeda*. Gravid uterus containing L1 larvae (L) (×10).

**FIG 5:**
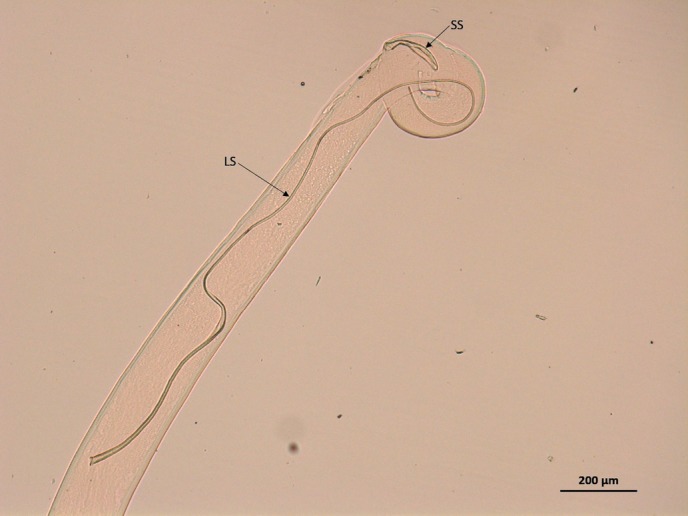
Light micrograph of male *Thelazia callipaeda*, caudal end containing two spicules of distinctly uneven length. LS, long spicule; SS, short spicule (×5).

All specimens possessed a buccal capsule, filariform oesophagus and transverse cuticular striations (Fig 2). The position of the vulva in the female specimens (all specimens examined from cases 1 and 2) was anterior to the oesophagus–intestinal junction (Fig 3). In addition, the female specimens examined from both cases 1 and 2 were identified as adults based on the presence of a gravid uterus containing first stage larvae (L1; Fig 4). A single male specimen (case 3) was curved at the caudal end, which contained two spicules of distinctly uneven length (Fig 5). The shorter spicule was crescent-shaped and the anterior extremity of the longer spicule was blunt and broadened.

## Molecular investigation via PCR and sequencing

DNA extraction from adult nematodes was performed on ethanol-preserved specimens using a QIAGEN DNeasy Blood and Tissue Kit following manufacturer’s recommendations (www.qiagen.com). Purified DNA was then assessed via PCR amplification using NTF/NTR primers designed to amplify the mitochondrial cytochrome c oxidase subunit 1 (*cox*1) gene of filarial and spirurid nematodes to yield a 689 bp product as described previously.^32^ To confirm both species and haplotype, PCR products were sequenced and compared with those available in the GenBank database, using Basic Local Alignment Search Tool (http://blast.ncbi.nlm.nih.gov/blast.cgi). The *cox*1 sequences obtained from nematodes collected from the three dogs were identical to the sequence of *T callipaeda* haplotype-1 (h1) (GenBank accession no. AM042549)

## Discussion

In all three instances dogs were known to have travelled to countries and regions, namely Romania, Italy and France, where *T callipaeda* populations to date have consisted exclusively of h1, as is the case across Europe.^10 28 32^ These three cases therefore demonstrate the risk of introducing *T callipaeda* to the UK through dogs being imported from and travelling to geographical locations where *T callipaeda* is known to be endemic. Furthermore, since *T callipaeda* is zoonotic and capable of infecting several other mammalian species, both people and cats should also be considered at risk of infection when travelling to such areas.^22 30^

While fulfilling the legal requirements of PETS, the coincidental administration of nematocides in the form of febantel and pyrantel in all cases and further treatment with piperazine and dichlorophen in case 3 are demonstrably unsuccessful in clearing *T callipaeda* infection. To date only milbemycin and moxidectin have been registered against ocular thelaziosis^6 8^ This represents a notable gap in the prophylactic measures present under PETS for this specific parasite, and we would encourage veterinarians to discuss the relevant treatment options with owners planning on travelling to or returning from such areas with their pets.

The clinical nature of cases 2 and 3 highlights the potential health and welfare issues of *T callipaeda* infection. Conversely, the subclinical carriage observed in case 1 is also of importance, as such infections may remain undiagnosed upon return to the UK. Gravid females were present in cases 1 and 2, which together with the presence of the vector in the UK highlights the potential for the parasite to become established in the UK.^33^ Similarly, the introduction of *T callipaeda* in a previously non-endemic region of Spain (La Vera) was thought to be due to hunting dogs travelling from endemic areas of Italy and France.^13^

*P variegata* is typically found in regions of deciduous (commonly oak) forest with greatest activity during warmer, humid periods.^26^ Ecological niche modelling for Europe in 2006 identified the southern parts of England as suitable habitat for *P variegata,* 26 with reports confirming its presence in Gloucestershire, Kent and Berkshire.^34–36^ Further investigation to establish the current distribution and population density of *P variegata* in the UK through a combination of entomological surveillance and ecological niche modelling with up-to-date climate data would help to determine the current risk of *T callipaeda* becoming endemic in the UK.

In summary, these three cases of canine ocular thelaziosis demonstrate the potential risks posed to the UK canine population from infection with *T callipaeda* through travel to and importation from parts of mainland Europe. Vigilance is therefore advised when examining travelled dogs. Although effective diagnostic tests and treatments are available, more can and should be done to prevent this zoonotic pathogen from becoming endemic in the UK.
